# Phased-array combination of 2D MRS for lipid composition quantification in patients with breast cancer

**DOI:** 10.1038/s41598-020-74397-y

**Published:** 2020-11-18

**Authors:** Vasiliki Mallikourti, Sai Man Cheung, Tanja Gagliardi, Nicholas Senn, Yazan Masannat, Trevor McGoldrick, Ravi Sharma, Steven D. Heys, Jiabao He

**Affiliations:** 1 Institute of Medical Sciences, School of Medicine, University of Aberdeen, Aberdeen, UK; 2 Department of Radiology, Royal Marsden Hospital, London, UK; 3 Breast Unit, Aberdeen Royal Infirmary, Aberdeen, UK; 4 Department of Oncology, Aberdeen Royal Infirmary, Aberdeen, UK

**Keywords:** Breast cancer, Medical imaging

## Abstract

Lipid composition in breast cancer, a central marker of disease progression, can be non-invasively quantified using 2D MRS method of double quantum filtered correlation spectroscopy (DQF-COSY). The low signal to noise ratio (SNR), arising from signal retention of only 25% and depleted lipids within tumour, demands improvement approaches beyond signal averaging for clinically viable applications. We therefore adapted and examined combination algorithms, designed for 1D MRS, for 2D MRS with both internal and external references. Lipid composition spectra were acquired from 17 breast tumour specimens, 15 healthy female volunteers and 25 patients with breast cancer on a clinical 3 T MRI scanner. Whitened singular value decomposition (WSVD) with internal reference yielded maximal SNR with an improvement of 53.3% (40.3–106.9%) in specimens, 84.4 ± 40.6% in volunteers, 96.9 ± 54.2% in peritumoural adipose tissue and 52.4% (25.1–108.0%) in tumours in vivo. Non-uniformity, as variance of improvement across peaks, was low at 21.1% (13.7–28.1%) in specimens, 5.5% (4.2–7.2%) in volunteers, 6.1% (5.0–9.0%) in peritumoural tissue, and 20.7% (17.4–31.7%) in tumours in vivo. The bias (slope) in improvement ranged from − 1.08 to 0.21%/ppm along the diagonal directions. WSVD is therefore the optimal algorithm for lipid composition spectra with highest SNR uniformly across peaks, reducing acquisition time by up to 70% in patients, enabling clinical applications.

## Introduction

Lipid composition is a central marker for the pathogenesis of breast cancer^1, 2^, the most commonly diagnosed cancer among women^3^. Conventional magnetic resonance spectroscopy (MRS) of stimulated echo acquisition mode (STEAM) with short echo time can detect lipid spectral peaks in the breast non-invasively on standard clinical scanners^4^, and further enhancement in specificity is valuable for clinical applications. Spectral editing methods of double quantum filtering (DQF), effectively suppress background signals, but only target a single metabolite, such as polyunsaturated fatty acids (PUFA) in 1D MRS^5^. The two dimensional (2D) MRS method of correlation spectroscopy (COSY)^6^ resolves lipid composition on a 2D map, but suffers from the dominant water signal and wide peak spread^7^. DQF-COSY, combining the strength of spectral editing and 2D MRS, allows unobscured identification of individual lipid resonances through sharp peak appearance and suppression of water contamination signals^8^. However, both the signal retention of only 25% in DQF-COSY^7^ and depleted lipids within breast tumours^5,9^ contribute to low signal to noise ratio (SNR), posing a challenge for accurate quantification. Since DQF-COSY collects a series of 1D spectra demanding a long acquisition time (typical scan time of 15–20 min)^10^, SNR improvement approaches beyond signal averaging are required for clinically viable applications.

Phased-array coils have been widely adopted in routine clinical practice, with signal combination algorithms developed to enhance SNR and reduce acquisition time^11,12^. Adaptively Optimised Combination (AOC)^13^, amongst current combination algorithms developed for 1D MRS (Table 1)^13–16^, is the optimal approach for spectra acquired in the brain using conventional MRS^13^ and PUFA spectra acquired in the breast using spectral editing MRS^17^. The SNR of a single spectral peak has been adopted as the common assessment criteria in the comparison of combination algorithms. However, lipid composition in 2D MRS is determined utilising multiple spectral peaks across the 2D map, demanding an algorithm with uniform improvement. In contrast to spectral editing MRS, DQF-COSY retains the presence of dominant metabolites, at reduced amplitude, for the estimation of sensitivities and phases of coil elements, potentially eliminating the need to acquire an additional reference spectrum.

**Table 1:** Summary of signal combination algorithms designed for 1D MRS.

Algorithms	Description
Equal weighting	Adding after aligning in phase
Signal weighting	Aligning in phase and weighting with the signal of reference peak
S/N weighting	Aligning in phase and weighting with the SNR of reference peak
S/N^2^ weighting	Aligning in phase and weighting with the signal to the noise squared (S/N^2^) of reference peak
nd-comb	Noise decorrelation using PCA, then aligning in phase and weighting the noise decorrelated data using the SNR of reference peak
WSVD	Noise decorrelation using PCA, then aligning in phase and weighting the noise decorrelated spectra using the first left singular vector obtained from the singular value decomposition of the noise decorrelated spectra
AOC	Phasing and weighting with the signal of reference peak multiplied by the inverted noise correlation matrix

*AOC* adaptively optimised combination, *CV* coefficient of variance, *nd-comb* noise decorrelated combination, *PCA* Principal Component Analysis, *WSVD* whitened singular value decomposition.

**Figure 1:**
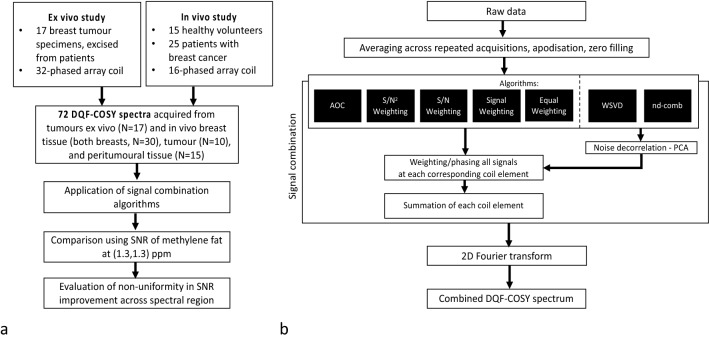
Diagram of study design and data processing. (**a**) Study design. Combination algorithms were evaluated on DQF-COSY spectra acquired from ex vivo and in vivo experiments by comparing the SNR. (**b**) Processing steps. Combination algorithms were applied on DQF-COSY spectra after signal averaging, apodisation and zero filling. AOC = adaptively optimised combination, nd-comb = noise decorrelated combination, WSVD = whitened singular value decomposition.

We hypothesise that AOC is the optimal algorithm to provide maximal SNR improvement uniformly across the 2D lipid composition spectrum in breast cancer. We therefore adapted current algorithms (Table 1), with a particular focus on noise decorrelated algorithms, for 2D MRS and applied on lipid composition spectra acquired using DQF-COSY. The combination algorithms were evaluated on spectra acquired from breast tumour specimens, healthy female volunteers and patients with breast cancer, with data from the tumour and the peritumoural adipose tissue (Fig. 1a). Each algorithm was implemented twice with weighting coefficients derived from the spectrum without water suppression (external reference, denoted by subscript “e”) and first signal of the DQF-COSY acquisition (internal reference, denoted by subscript “i”) (Fig. 1b). The non-uniformity of SNR improvement across spectral peaks (Table 2) and the direction of non-uniformity was additionally evaluated. The non-uniformity was defined as the coefficient of variance of SNR improvement across spectral peaks. The direction of non-uniformity was quantified as the slopes along the diagonal (bias along the frequency axes) and the off-diagonal (bias along the encoding axes) on a plane regressed to the SNR improvement at the spectral location of each peak.

**Table 2:** Peaks in 2D DQF-COSY spectra from breast.

Spectral peaks	Locations (F_2_, F_1_) (ppm)
Methyl protons	(0.9, 0.9)
Methylene protons	(1.3, 1.3)
Methylene protons $\beta$ to COO	(1.6, 1.6)
Allylic methylene protons	(2.1, 2.1)
Methylene protons $\alpha$ to COO	(2.4, 2.4)
Diallylic methylene protons	(2.8, 2.8)
Glycerol methylene protons	(4.3, 4.3)
Olefinic protons	(5.3, 5.3)
Unsaturated fatty acid cross peak	(5.3, 2.8)
Unsaturated fatty acid cross peak	(5.3, 2.1)
Unsaturated fatty acid cross peak	(2.8, 5.3)
Unsaturated fatty acid cross peak	(2.1, 5.3)

## Results

### SNR among algorithms

**Figure 2:**
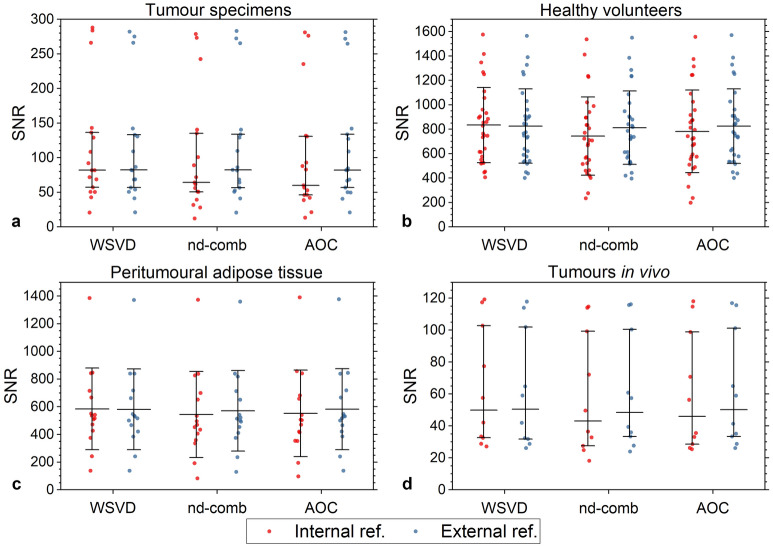
Comparison of the combination algorithms on DQF-COSY spectra using external and internal references. SNR of DQF-COSY spectra from (**a**) breast tumour specimens, (**b**) healthy volunteers, (**c**) peritumoural adipose tissue, and (**d**) tumours in vivo, using AOC, nd-comb, and WSVD. Median and interquartile range are shown for non-normally distributed data while mean and standard deviation are shown for normally distributed data. External and internal references were derived from the unsuppressed water spectrum and the spectrum of first signal (increment) in DQF-COSY respectively.

**Figure 3:**
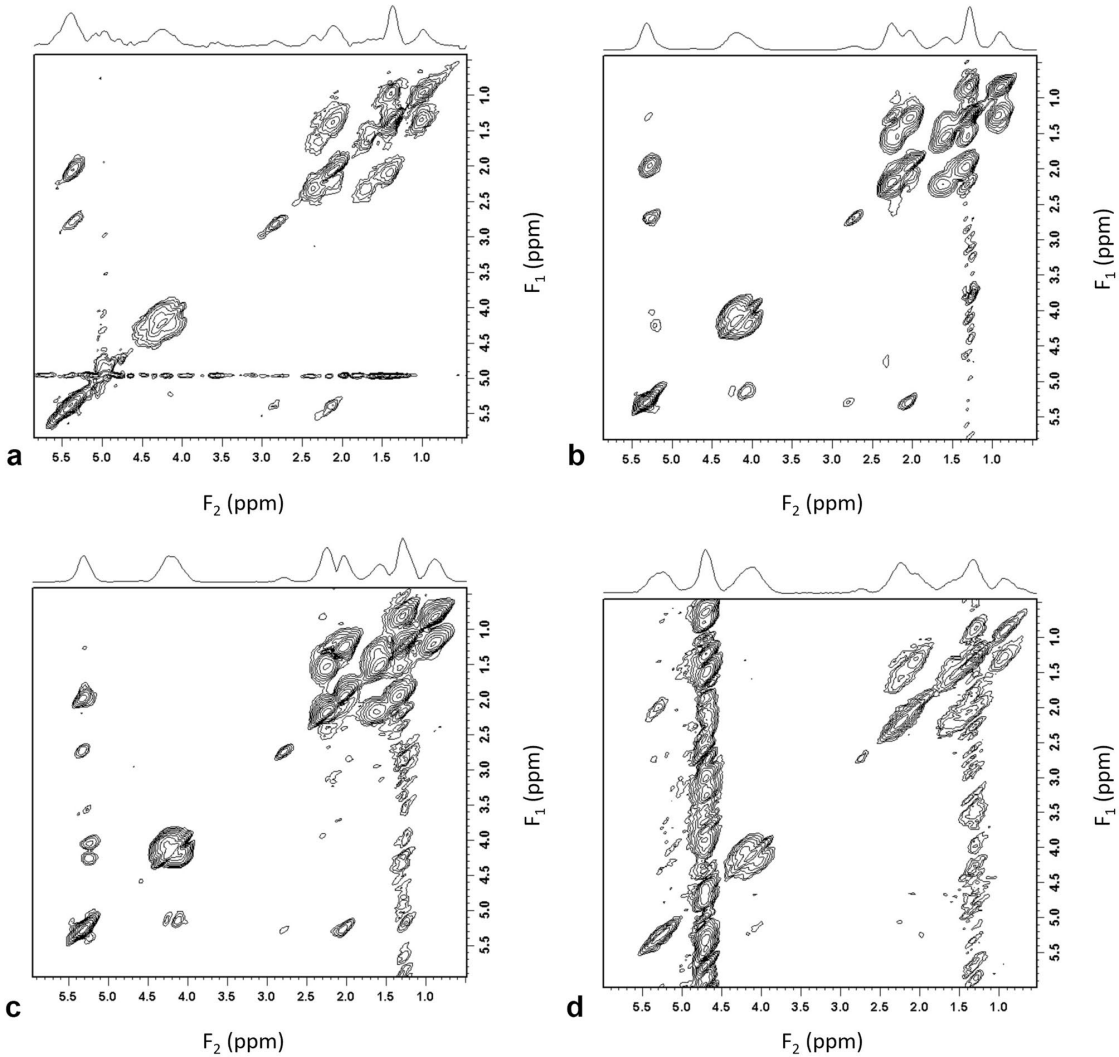
DQF-COSY combined spectra. DQF-COSY combined spectra using WSVD_i_ from (a) a specimen of invasive carcinoma, grade III, (**b**) the left breast of a healthy volunteer, (**c**) peritumoural adipose tissue from a patient with invasive carcinoma, grade III, and (**d**) an invasive carcinoma, grade III from a patient.

For tumour specimens, the SNR from WSVD_i_ (median 81.9, interquartile range 57.2–136.4) was comparable against WSVD_e_, but significantly higher than the other approaches (Table 3, Fig. 2a). For healthy volunteers, WSVD_i_ yielded the highest SNR (833.6 ± 308.6) with statistical significance (Table 3, Fig. 2b). For peritumoural adipose tissue in patients, the SNR from WSVD_i_ (584.6 ± 294.7) was comparable against AOC_e_, but significantly higher than the other approaches (Table 3, Fig. 2c). For tumours in vivo, the SNR from WSVD_i_ (49.8, 32.6–102.8) was significantly higher (p = 0.028) than nd-comb_i_ (43.1, 27.5–99.3), but comparable against other approaches (Table 3, Fig. 2d). Linear algorithms gave lower SNR compared with the noise decorrelated algorithms, and are presented only for information purposes (Table 3). WSVD_i_ improved the SNR by 53.3% (40.3–106.9%) in specimens, 84.4 ± 40.6% in volunteers, 96.9 ± 54.2% in peritumoural adipose tissue and 52.4% (25.1–108.0%) in tumours in vivo, reducing the acquisition time by 50–70% in tumour and adipose tissue respectively. The combined 2D spectra using WSVD_i_ from a specimen, volunteer, peritumoural tissue, and tumour in vivo are shown in Fig. 3.

**Table 3:** SNR of (1.3, 1.3) ppm in 2D lipid composition spectra using DQF-COSY.

	Algorithms	Tumour specimens (N = 17)	P-value	Healthy volunteers (N = 15, 30 voxels)	P-value	Patients Peritumoural adipose tissue (N = 15)	P-value	Patients Tumours in vivo (N = 10)	P-value
External reference	Equal	59.5 (41.6–105.8)		469.5 ± 127.7		309.9 ± 112.5		37.8 (19.7–57.0)	
	Signal	75.4 (52.3–131.3)		621.4 ± 239.6		444.6 ± 215.9		42.9 (25.8–81.0)	
	S/N	76.9 (53.2–133.0)		637.4 ± 235.2		462.2 ± 219.2		46.1 (25.2–82.4)	
	S/N^2^	78.2 (53.3–133.4)		645.0 ± 229.1		472.4 ± 218.4		47.7 (24.8–81.9)	
	WSVD	82.3 (56.8–133.1)	0.246	825.6 ± 304.5	0.003	581.1 ± 291.6	0.022	50.5 (31.8–101.9)	0.241
	nd-comb	82.4 (56.7–133.8)	0.049	812.1 ± 300.7	< 0.001	570.7 ± 291.0	< 0.001	48.5 (33.4–100.4)	0.575
	AOC	82.5 (56.8–133.9)	0.003	825.7 ± 305.2	0.003	582.2 ± 293.2	0.100	50.2 (33.4–101.3)	0.575
Internal reference	Equal	39.5 (30.8–103.9)		457.4 ± 148.4		311.4 ± 154.6		37.4 (18.2–57.0)	
	Signal	54.1 (41.0–129.9)		609.8 ± 248.5		433.2 ± 230.8		41.2 (23.9–82.9)	
	S/N	57.5 (41.9–131.8)		624.9 ± 244.5		449.2 ± 237.2		43.6 (24.4–82.5)	
	S/N^2^	60.0 (41.6–132.2)		630.0 ± 240.4		482.5 ± 239.1		44.9 (24.6–81.4)	
	WSVD	81.9 (57.2–136.4)	–	833.6 ± 308.6	–	584.6 ± 294.7	–	49.8 (32.6–102.8)	–
	nd-comb	64.2 (50.6–135.2)	< 0.001	743.6 ± 319.8	0.001	543.6 ± 310.7	0.007	43.1 (27.5–99.3)	0.028
	AOC	60.0 (46.1–130.8)	< 0.001	781.4 ± 338.1	0.005	552.6 ± 312.9	0.034	46.0 (28.6–98.9)	0.059

The median SNR and interquartile range are shown for non-normally distributed data while mean and standard deviation are shown for normally distributed data. P-value represents the comparison on SNR between the noise decorrelated algorithms using repeated measures ANOVA or Wilcoxon signed-rank tests. The SNR of the linear algorithms is also reported for reference purposes. Results are presented with WSVD_i_ as a reference for comparison.

*AOC* adaptively optimised combination, *CV* coefficient of variance, *nd-comb* noise decorrelated combination, *WSVD* whitened singular value decomposition.

### Non-uniformity of SNR improvement across spectrum

**Figure 4:**
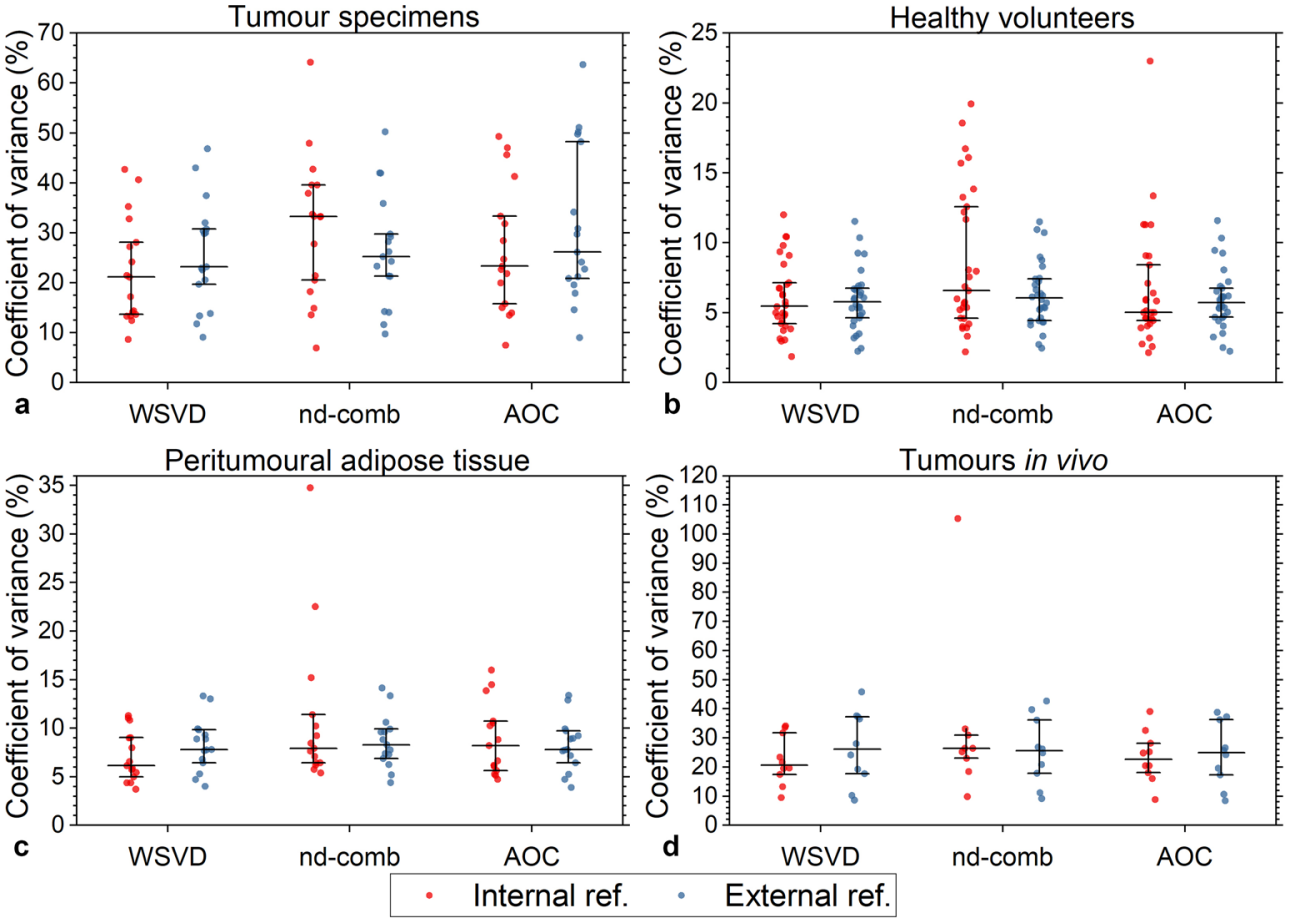
Comparison of non-uniformity of SNR improvement across the 2D spectrum in DQF-COSY. The non-uniformity of SNR improvement using the noise decorrelated algorithms of AOC, nd-comb, and WSVD is shown for DQF-COSY spectra in (**a**) breast tumour specimens, (**b**) healthy volunteers, (**c**) peritumoural adipose tissue, and (**d**) tumours in vivo. The non-uniformity of improvement was defined as the coefficient of variance of SNR improvement across all spectral peaks in a 2D spectrum. The error bars show the median and interquartile range. Results using both internal and external references are shown. Data with negative SNR improvement were excluded (1 dataset from a healthy volunteer using AOC_i_, 1 dataset from a healthy volunteer using nd-comb_i_, and 1 dataset from a tumour in vivo using nd-comb_i_).

**Figure 5:**
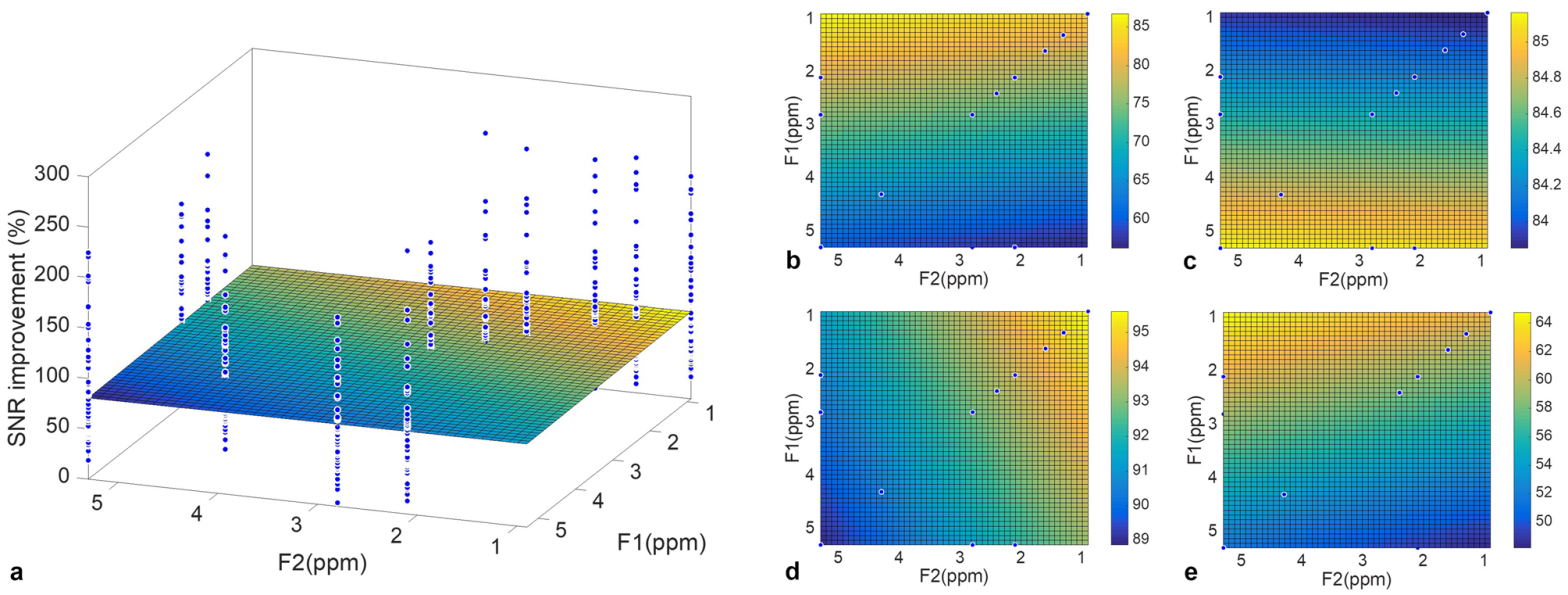
SNR improvement distribution in a 3D plane for DQF-COSY. (**a**) The SNR improvement obtained from WSVD_i_ is plotted at the spectral location of each peak in a 3D scatter plot. Colour maps showing the direction and degree of non-uniformity of SNR improvement are shown for WSVD_i_ for (**b**) tumour specimens, (**c**) healthy volunteers, (**d**) peritumoural adipose tissue, and (**e**) tumours in vivo. The dots show the location of diagonal and cross peaks. The colour bar shows the SNR improvement.

For tumour specimens, WSVD_i_ had significantly the lowest non-uniformity (21.1%, 13.7–28.1%, p < 0.05) (Table 4, Fig. 4a), with higher improvement towards low frequencies along diagonal direction (slope of − 3.79%/ppm) and mixing encoding (t_1_/F_1_) along off-diagonal direction (slope of 4.90%/ppm) (Table 4, Fig. 5a,b). For healthy volunteers, WSVD_i_ had significantly lower non-uniformity (5.5%, 4.2–7.2%) compared against nd-comb_i_ (6.6%, 4.6–12.9%, p < 0.001), but no significant differences against the other algorithms (Table 4, Fig. 4b). Higher improvement was found towards high frequencies along diagonal direction (slope of 0.21%/ppm) and reading encoding (t_2_/F_2_) along off-diagonal direction (slope of − 0.18%/ppm) (Table 4, Fig. 5a,c)_._ For peritumoural adipose tissue, WSVD_i_ had significantly lower non-uniformity (6.1%, 5.0–9.0%) than AOC_i_ (8.2%, 5.6–10.7%), nd-comb_i_ (7.9%, 6.4–11.4%) and nd-comb_e_ (8.3%, 6.9–9.9%), but no significant difference from the other algorithms (Table 4, Fig. 4c). Higher improvement was found towards low frequencies along diagonal direction (slope of − 1.08%/ppm) and reading encoding (t_2_/F_2_) along off-diagonal direction (slope of − 0.30%/ppm) (Table 4, Fig. 5a,d). For tumours in vivo, there were no significant differences in non-uniformity from WSVD_i_ (20.7%, 17.4–31.7%) against the other algorithms (Table 4, Fig. 4d), with higher improvement towards low frequencies along diagonal direction (slope of − 1.1%/ppm) and mixing encoding (t_1_/F_1_) along off-diagonal direction (slope of 2.68%/ppm) (Table 4, Fig. 5a,e).

**Table 4:** Non-uniformity of SNR improvement in 2D lipid composition spectra using DQF-COSY.

	Algorithms	Tumour specimens (N = 17)				Healthy volunteers (N = 15, 30 voxels)				Patients Peritumoural adipose tissue (N = 15)				Patients Tumours in vivo (N = 10)			
		Slopes (%/ppm)		CV (%)	P-value	Slopes (%/ppm)		CV (%)	P-value	Slopes (%/ppm)		CV (%)	P-value	Slopes (%/ppm)		CV (%)	P-value
		Diag	Off diag			Diag	Off diag			Diag	Off diag			Diag	Off diag		
External reference	WSVD	0.53	2.72	23.2 (19.7–30.8)	0.044	− 0.07	− 0.43	5.8 (4.6–6.7)	0.491	− 1.26	− 0.47	7.8 (6.4–9.8)	0.139	− 0.99	1.54	26.1 (17.7–37.2)	0.139
	nd-comb	0.60	2.59	25.2 21.3–29.8)	0.022	− 0.10	− 0.42	6.0 (4.4–7.4)	0.959	− 1.34	− 0.47	8.3 (6.9–9.9)	0.445	− 1.07	1.17	25.5 (17.9–36.1)	0.445
	AOC	− 0.29	2.46	26.2 (20.9–8.3)	0.001	− 0.06	− 0.45	5.7 (4.7–6.8)	0.558	− 1.27	− 0.48	7.8 (6.4–9.7)	0.575	− 1.03	0.92	25.0 (17.3–36.3)	0.575
Internal reference	WSVD	− 3.79	4.90	21.1 13.7–28.1)	–	0.21	− 0.18	5.5 (4.2–7.2)	–	− 1.08	− 0.30	6.1 (5.0–9.0)	–	− 1.1	2.68	20.7 (17.4–31.7)	–
	nd-comb	− 1.01	1.66	33.3 (20.5–39.6)	0.006	− 0.03	− 0.28	6.6 (4.6–12.6)	< 0.001	− 0.99	− 0.88	7.9 (6.4–1.4)	0.374	− 1.2	2.3	26.4 (23.0–30.9)	0.374
	AOC	− 0.65	2.02	23.3 (15.8–33.3)	0.002	− 0.11	− 0.24	5.0 (4.4–8.4)	0.567	− 0.90	− 0.48	8.2 (5.6–0.7)	0.575	− 3.4	0.41	22.7 (18.1–28.2)	0.575

Data for CV are medians (interquartile range). Slope along the diagonal is positive from (0.9, 0.9) ppm to (5.3, 5.3) ppm. Slope along the off diagonal is positive from reading t_2_/F_2_ at (0.9, 5.3) ppm to mixing t_1_/F_1_ at (5.3, 0.9) ppm. P-value represents the comparison on CV values between the noise decorrelated algorithms using Wilcoxon signed-rank tests. Results are presented with WSVD_i_ as a reference for comparison. Data with negative SNR improvement were excluded (1 dataset from a healthy volunteer using AOC_i_, 1 dataset from a healthy volunteer using nd-comb_i_, and 1 dataset from a tumour in patients using nd-comb_i_). AOC = adaptively optimised combination, CV = coefficient of variance, nd-comb = noise decorrelated combination, WSVD = whitened singular value decomposition.

## Discussion

In this work, current combination algorithms, designed for 1D MRS, were adapted and evaluated for lipid composition spectra from breast acquired using 2D MRS, with a particular focus on noise decorrelation algorithms. WSVD_i_ was identified as the most effective signal combination approach in 2D MRS, instead of AOC, the optimal algorithm for 1D MRS^13,17^. WSVD_i_ provided maximal SNR improvement in patients (97% in peritumoural adipose tissue, 52% in tumour) and low non-uniformity of 6% and 21% respectively. WSVD_i_, eliminating the need for acquiring an additional reference spectrum (typically scan time of 2 min), reduces scan time by 50–70% from 17 to 8 min in tumour and 5 min in peritumoural adipose tissue.

Noise decorrelation algorithms outperformed all linear algorithms substantially through the cancellation of correlated noise, as found in 1D MRS studies^13,17^. WSVD performance was not degraded in low SNR spectra acquired from the tumours, in contrast to 1D spectra reported in previous studies^17,18^. In high SNR spectra from adipose tissue, WSVD and AOC yielded comparable SNR and outperformed nd-comb, as observed in 1D PUFA spectra^17^. Among the linear algorithms, S/N^2^ Weighting had the best SNR performance, in line with the 1D MRS studies^14,17^.

External reference methods (WSVD_e_, AOC_e_ and nd-comb_e_) showed comparable SNR ex vivo, with the performance of nd-comb_e_ degraded in vivo due to the larger variation in coil weightings associated with the voxel location away from the isocentre^14,15,19^. For internal reference, WSVD_i_ outperformed AOC_i_ and nd-comb_i_. External reference, with higher SNR than internal reference, is expected to provide more accurate weighting coefficients and in turn higher SNR of combined spectra^20^, as observed in AOC and nd-comb. However, WSVD is less sensitive to the SNR of the reference spectrum as observed in this work, with weighting coefficients generated from the entire spectrum^16^ instead of a dominant peak, as in AOC^13^ and nd-comb^15^.

WSVD_i_, in addition to providing maximal SNR, eliminates the need for the acquisition of a reference spectrum, reducing scan time by approximately 2 min. WSVD_i_ improved the SNR by 97% in peritumoural adipose tissue and 52% in tumours in vivo, allowing the acquisition of a lipid composition spectrum in less than 8 min in patients instead of 17 min (15 min for lipid composition spectrum and 2 min for reference spectrum).

WSVD_e_ and WSVD_i_ showed better or comparable non-uniformity against other external and internal reference algorithms, with similar performance between WSVD_e_ and WSVD_i_. WSVD_i_ had non-uniformity of 21% in specimens, 5% in volunteers, 6% in peritumoural adipose tissue and 21% in tumours in vivo, with the variation in non-uniformity reflecting the effects of noise on SNR improvement. The slope along the diagonals further confirmed the observation of non-uniformity, as higher slope (bias in SNR improvement) was associated with lower SNR (observed in spectra from tumours) and lower slope was associated with higher SNR (observed in spectra from adipose tissue). The magnitude of the slop was small in adipose tissue (0.21%/ppm and 0.18%/ppm in healthy volunteers and 1.08%/ppm and 0.30%/ppm in peritumoural adipose tissue), indicating negligible changes of SNR improvement of 0.92% and 0.79% in healthy volunteers and 4.8% and 1.32% in peritumoural adipose tissue at a maximal frequency gap between peaks of interest (0.9 ppm to 5.3 ppm). However, the slopes found in tumours were noticeably higher with higher improvement towards low frequencies and mixing encoding directions, likely due to the signal elevation closer to contamination water signal stripe along the mixing encoding direction (Fig. 3d). Hence, WSVD_i_ provides minimal non-uniformity for SNR improvement across lipid composition spectra.

DQF-COSY^8^, similar to STEAM^21^, is composed of three 90° RF pulses, allowing a short echo time and a minimal chemical shift displacement. DQF-COSY, different from STEAM, modulates the evolution time ${\mathrm{t}}_{1}$ for 2D spectral encoding and incorporates quantum coherence pathway selection gradients to suppress background signal, allowing enhanced specificity at the expense of a portion of SNR^8^. DQF-COSY directly resolves monounsaturated fatty acids (MUFA) and PUFA through J-coupling sensitivity^8^, while STEAM instead demands a mathematical model^22^. All lipid peaks could be well detected using conventional 1D MRS under reasonable water suppression^23^, while DQF-COSY may have a big advantage for the detection of lipid peaks at 4.1 ppm and 4.25 ppm under challenging conditions for water suppression. Hence, DQF-COSY, supported by its intrinsic minimal chemical shift displacement, high specificity and SNR enhancement from a phased-array combination approach may help studying small lesions or area of interest.

The extensive experiments on ex vivo tumour specimens, healthy participants and patients (over 70 datasets from clinical population) encompassed a wide range of physiological environments encountered in a clinical setting. The acquisition voxel was adjusted to the size of the tumour in diseased breast and standardised to 2 $\times$ 2 $\times$ 2 cm^3^ in healthy breast, allowing investigation in both low and high SNR conditions. The comparison among algorithms was comprehensive, covering both internal and external references, with outcome measures extended beyond conventional SNR to non-uniformity. Both standard clinical hardware (scanner and coil) and routine patient imaging procedures were adopted to ensure the immediate clinical translation of the research findings. This study was limited to a single scanner and two different coils, and multi-centre studies on scanners and coils from a range of vendors are required before wider clinical adoption. Patients with invasive breast carcinoma were studied in this work to reduce the confounding factor of experimental setup, however larger patient cohorts with other phenotypes of breast cancer should be investigated in the future.

WSVD, the most effective combination algorithm for 2D MRS, can enhance the sensitivity of inconspicuous lipid constituents found in tumours and accelerate the acquisition in adipose tissue. The extension of single voxel 2D MRS into chemical shift imaging (CSI) of 2D MRS allows the investigation of spatial distribution of lipid composition in tumour but is limited by the voxel size and a demanding acquisition time proportional to the number of voxels acquired. WSVD can potentially allow smaller voxel sizes and reduce acquisition time through trading the enhanced SNR, allowing the investigation of spatially heterogeneous response to neoadjuvant chemotherapy in breast cancer^24^. CSI of 2D MRS, with further support from compressed sensing, can achieve direct lipid composition mapping of the entire breast, for the early detection and prevention of breast cancer, without the need of a mathematical model of lipid amplitudes based on the empirical assumptions made in the Dixon method^25^. However, further investigation is needed to consider the spatial variability in the coil sensitivity and the combination of WSVD with compressed sensing for CSI of 2D MRS.

In conclusion, WSVD_i_, instead of AOC, is the optimal approach for processing lipid composition spectra acquired using 2D MRS on phased-array coils from the breast. WSVD_i_ not only provides maximal SNR improvement without the need of additional reference spectra, but also delivers consistent improvement across lipids with high uniformity. With improved SNR, the acquisition can be achieved at a clinically viable time of 8 min (instead of 17 min), enabling the routine clinical assessment of lipid composition.

## Methods

A total of 72 lipid composition spectra were acquired using DQF-COSY from excised human breast tumour specimens, healthy female volunteers and patients with breast cancer (Fig. 1a). The ex vivo study was approved by the North West—Greater Manchester East Research Ethics Committee (REC reference: 16/NW/0032). The in vivo studies were approved by the North of Scotland Research Ethics Service (REC reference: 16/NS/0077) and the London—Central Research Ethics Service (REC reference: 17/LO/1777). All experiments were conducted in accordance with the Declaration of Helsinki guidelines and all participants provided written informed consent prior to the study. All scans were performed on a 3 T clinical MRI scanner (Achieva TX, Philips Healthcare, Best, Netherlands) using a body coil for uniform transmission.

### Ex vivo study

Seventeen female patients (mean age 61 years, age range 42–78 years) with invasive carcinoma (eight grade II and nine grade III), without prior hormonal therapy or chemotherapy and a tumour size greater than 10 mm in diameter were enrolled. The freshly excised whole tumour at surgery was immediately scanned before formalin treatment using a 32-element phased-array receiver coil for signal detection. Clinical standard T_1_-weighted and T_2_-weighted anatomical images were acquired for voxel localisation. 2D spectra of lipid composition were acquired using DQF-COSY^8^ with repetition time (TR) of 552 ms, initial echo time (TE) of 25 ms, a t_1_ increment of 1 ms, 256 increments (mixing encoding t_1_ time domain axis, F_1_ frequency domain axis), 256 sampling points (reading encoding t_2_ time domain axis, F_2_ frequency domain axis), 4 repeats per increment, spectral bandwidth of 1000 Hz, and DQF gradients of 30/40/100 ms mT/m. Reference spectra without water suppression were acquired using single voxel PRESS sequence^26^ with TR/TE of 1250/144 ms, 1024 data points, spectral bandwidth of 2000 Hz and 16 averages. The voxel was positioned to cover the whole tumour, with a voxel volume ranging from 2.7 to 16.5 cm^3^ according to tumour size.

### In vivo study

Fifteen healthy female volunteers (mean age 66 years, age range 58–76 years) without previous breast cancer or family history of breast cancer participated in the study. Fifteen patients (mean age 63 years, age range 53–71 years, seven grade II and eight grade III) and a further ten patients (mean age 52 years, age range 36–63 years, one grade II and nine grade III) with invasive carcinoma were enrolled into the study for the acquisition of lipid composition spectra from peritumoural adipose tissue and tumour respectively. Patients with a tumour size greater than 10 mm, without prior chemotherapy or hormonal therapy, and no conditions contraindicative to MRS were eligible. All participants were scanned in the prone position as clinical routine practice using a 16-element phased-array breast receiver coil for signal detection. Standard sagittal T_1_-weighted anatomical images, axial T_2_-weighted anatomical images and diffusion weighted images were acquired for voxel localisation. 2D spectra of lipid composition were acquired using DQF-COSY^8^ with TR of 552 ms, initial TE of 25 ms, a t_1_ increment of 1 ms, 256 increments (mixing encoding t_1_ time domain axis, F_1_ frequency domain axis), 256 sampling points (reading encoding t_2_ time domain axis, F_2_ frequency domain axis), 2 repeats per increment, spectral bandwidth of 1000 Hz, and DQF gradients of 30/40/100 ms mT/m. Reference spectra without water suppression were acquired using single voxel PRESS sequence^26^ with TR/TE of 1250/144 ms, 1024 data points, spectral bandwidth of 2000 Hz and 16 averages. In healthy volunteers, data were acquired from both breasts and the voxel size was set to 2 $\times$ 2 $\times$ 2 cm^3^ containing primarily the adipose tissue. In patients, the voxel covering the tumour had a volume ranging from 2.2 to 21 cm^3^ for tumours in vivo (10 patients), while a voxel of 2 $\times$ 2 $\times$ 2 cm^3^ was positioned at 1 cm from the tumour for peritumoural adipose tissue (15 patients).

### Data processing

All the algorithms were developed in MATLAB (MathWorks, Natick, MA, USA) with the processing flowchart shown in Fig. 1b. The raw data were averaged across repeated acquisitions before signal combination. The averaged signal, organised as a 2D map based on t_1_ and t_2_ time domain axes for each coil element, was subsequently apodised using squared sine bell along both time domain axes and zero filled to 512 $\times$ 512 points. The reference spectrum without water suppression was used as external reference with the maximum peak (either water or lipid) as target metabolite^17^ while the first t_1_ increment of the DQF-COSY acquisition was used as internal reference with the maximum peak in frequency domain (either residual water or lipid) as the target metabolite. Both external (denoted by subscript “e”) and internal (denoted by subscript “i”) weighting coefficients, containing weights and phase, were computed using external and internal references respectively for each dataset and for each algorithm. The weighting coefficient derived for a coil element was applied to the apodised and zero filled signals at the corresponding coil element. The combined 2D time domain signal was the summation across all the coil elements, and the combined 2D spectrum was subsequently derived using 2D Fast Fourier transform.

The SNR of a spectral peak (Table 2) was computed as the peak height in the magnitude spectral map divided by the standard deviation of the real part of the noise in the square region covering (F_1_: 6.9–8.4, F_2_: 6.0–7.5) ppm^27^, with the overall SNR quantified at (1.3, 1.3) ppm and SNR improvement referenced to Equal Weighting algorithm^27^. The non-uniformity of SNR improvement across the 2D spectrum was subsequently derived as the coefficient of variance (standard deviation divided by the mean) of the SNR improvement across all the spectral peaks^19^. A 3D scatter plot of SNR improvement at the spectral location of the peak was then created (Fig. 5a). A plane was subsequently regressed onto the 3D scatter plot to derive the direction and degree of bias in frequency along the diagonal (low at (0.9, 0.9) ppm to high at (5.3, 5.3) ppm) and in encoding along the off diagonal (reading t_2_/F_2_ at (0.9, 5.3) ppm to mixing t_1_/F_1_ at (5.3, 0.9) ppm).

### Statistical analysis

Statistical analysis was performed in SPSS (Release 24.0, SPSS Inc., Chicago, USA). Shapiro–Wilk test was performed on the SNR and non-uniformity to assess if the distribution was normal. The SNR across noise decorrelated algorithms was compared using a one-way ANOVA with repeated measures and a Wilcoxon signed-rank test for normally and non-normally distributed data respectively. The SNR of the linear algorithms was also reported for reference purposes. Data with negative SNR improvement for the calculation of the non-uniformity were excluded (1 healthy volunteer using AOC_i_, 1 healthy volunteer using nd-comb_i_, and 1 tumour in vivo using nd-comb_i_). A p-value < 0.05 was considered statistically significant.

## Data Availability

The datasets generated during and/or analysed during the current study are available from the corresponding author on reasonable request.
